# Bone Mesenchymal Stem Cell-Derived Small Extracellular Vesicles Ameliorated Lipopolysaccharide-Induced Lung Injury Via the miR-21-5p/PCSK6 Pathway

**DOI:** 10.1155/2023/3291137

**Published:** 2023-10-28

**Authors:** Bo Cai, Weidong Song, Song Chen, Jie Sun, Rui Zhou, Zhen Han, Jian Wan

**Affiliations:** Department of Emergency and Critical Care Medicine, Shanghai Pudong New Area People's Hospital, No. 490 Chuansha South Road, Pudong New Area, 201299, Shanghai, China

## Abstract

Acute lung injury (ALI) is a life-threatening disease that currently lacks a cure. Although stem cell-derived small extracellular vesicles (sEVs) have shown promising effects in the treatment of ALI, their underlying mechanisms and responsible components have yet to be identified. Proprotein convertase subtilisin/kexin type 6 (PCSK6) is a gene involved in inflammation and a potential target of miR-21-5p, a microRNA enriched in stem cell-derived sEVs. The current study investigated the role of PCSK6 in lipopolysaccharide (LPS)-induced ALI and its interaction with miR-21-5p. Notably, our results showed that PCSK6 expression was positively correlated with LPS stimulation. Knockdown of PCSK6 ameliorated LPS-induced inhibition of proliferation and upregulation of permeability in human BEAS-2B cells, whereas PCSK6 overexpression displayed the opposite effects. BEAS-2B cells were able to actively internalize the cocultured bone mesenchymal stem cell (MSC)-derived sEVs (BMSC-sEVs), which alleviated the cell damage caused by LPS. Overexpressing PCSK6, however, eliminated the therapeutic effects of BMSC-sEV coculture. Mechanistically, BMSC-sEVs inhibited PCSK6 expression via the delivery of miR-21-5p, which is directly bound to the PCSK6 gene. Our work provides evidence for the role of PCSK6 in LPS-induced ALI and identified miR-21-5p as a component of BMSC-derived sEVs that suppressed PCSK6 expression and ameliorated LPS-induced cell damage. These results reveal a novel molecular mechanism for ALI pathogenesis and highlight the therapeutic potential of using sEVs released by stem cells to deliver miR-21-5p for ALI treatment.

## 1. Introduction

Acute lung injury (ALI) causes inflammatory damage to lung endothelial and epithelial barriers, inducing increased capillary permeability, neutrophil overactivation, transepithelial migration, and induction of pro-inflammatory molecules. ALI is triggered by multiple factors, including sepsis, acid aspiration, and E-cigarette/vaping [1]. Despite the already high incidence rates of ALI, the current COVID-19 pandemic has caused a drastic surge among ALI cases in SARS-CoV-2-infected patients [2], raising serious health concerns. ALI can cause pulmonary fibrosis and impair the lung's function to provide oxygen, which can eventually lead to dangerously low blood oxygen levels and cause death in several weeks [1]. Nowadays, ALI has been considered a severe threat to the health of people worldwide, with no therapeutic drugs available for its treatment. Despite the current use of ventilator management as a treatment strategy for ALI, in addition to high-quality care and monitoring, hospital mortality rates of ALI can still reach as high as ∼40% [3]. Thus, a more efficient therapeutic approach is urgently needed.

Mesenchymal stem cells (MSCs) are multipotent stem cells that differentiate into various lineages, like adipogenic, chondrogenic, and osteogenic lineages. As demonstrated by previous studies, stem cell therapy can efficiently reduce the frequency of death in animal ALI models, implying its potential as a novel therapeutic strategy for ALI [4]. Several mechanisms have been suggested or speculated to explain the therapeutic effects of stem cells in ALI [5]. For instance, stem cells may differentiate into lung cells to replace damaged ones, inhibit destructive inflammatory responses, and facilitate the repair of the lungs and other injured organs. Small extracellular vesicles (sEVs, with the diameter smaller than 200 nm) are extracellular vesicles secreted by cells and contain numerous constituents like DNA, RNA, and proteins (with CD63 and CD81 being the two most frequently identified ones) [6]. sEVs serve as a route for intercellular communication, in which sEVs secreted by one cell can be associated with and/or internalized by another cell, allowing the latter to receive the contained biological information [7]. For instance, sEVs secreted by T cells can bind to dendritic cells and induce their apoptosis. Moreover, sEVs released by MSCs have demonstrated superior utility in cancer diagnostics and the treatment of myocardial infarction, cutaneous wound healing, hepatic injury, and ALI [6, 8]. Meanwhile, encouraging results have been obtained with the use of MSC-derived sEVs for the treatment of lung injury in the context of the COVID-19 pandemic. Allogenic MSCs and MSC-derived sEVs significantly reduce the risk of alveolar inflammation and other pathological conditions related to obvious lung injuries [9]. MSCs and MSC-derived sEVs have been developed for the treatment of COVID-19, which have yielded promising outcomes [10]. However, the detailed molecular mechanism through which sEVs contribute therapeutically to ALI remains largely unclear.

One of the mechanisms through which sEVs regulate the targeted cell's behavior involves the release of microRNAs (miRNAs), which can disrupt or alter protein expression in the targeted cell via post-transcriptional gene regulation. Multiple MSC-derived miRNAs in sEVs have been identified to mediate cancer signaling and facilitate tumor development, metastasis, and drug resistance. Interestingly, recent studies have shown that some miRNAs that are potentially expressed in sEVs can alleviate ALI. For instance, miR-490, an miRNA that exists in the sEVs of mast cells [11], has been reported to alleviate sepsis-induced ALI in newborn mice, whereas miR-17, which can be found in human umbilical cord MSC-derived sEVs [12], had been shown to suppress inflammatory responses in lipopolysaccharide (LPS)-induced ALI [13]. Moreover, miR-21-5p, another microRNA highly expressed in MSC-derived sEVs [14], had been reported to alleviate hyperoxic ALI in rats [15]. Interestingly, through a database search, we discovered a potential gene target of miR-21-5p, namely proprotein convertase subtilisin/kexin type 6 (PCSK6), which prompted us to test our hypothesis of whether MSC-derived sEVs can deliver their contained miR-21-5p to lung epithelial cells and elicit therapeutic effects. PCSK6 is a member of the proprotein convertase family that cleaves and regulates the activity of numerous proteins, suggesting its importance in several physiological and pathological processes like embryogenesis, tumor progression, and cardiovascular diseases. Importantly, given evidence showing a positive correlation between PCSK6 and inflammation-related genes, inhibiting PCSK6 can provide protection against rheumatoid arthritis, supporting its involvement in inflammatory responses. However, the role of PCSK6 in ALI-related inflammation has yet to be studied, and its relationship with miR-21-5p in the context of physiology and pathology also remains unclear.

Here, we provide evidence suggesting that the newly discovered miR-21-5p/PCSK6 pathway could be an underlying mechanism for the therapeutic effects of bone MSC-derived sEVs (BMSC-sEVs) in ALI. We found that suppressing PCSK6 directly inhibited inflammatory responses in bronchial epithelial cells, whereas miR-21-5p contained in BMSC-sEVs could inhibit PCSK6 expression in the ALI cell model. Together, our findings highlight the potential and utility of miRNAs in BMSC-sEVs for the treatment of ALI.

## 2. Material and Methods

### 2.1. Cell Culture

Normal human BEAS-2B cells were acquired from the Shanghai Biology Institute (Shanghai, P.R. China). Third-generation cells were used in subsequent experiments. Human BMSCs were obtained from Shang Hai Ze Ye Biotechnology (Shanghai). The cells were grown in DMEM (Trueline, Kaukauna, WI, USA) media supplemented with a 10% sEV-depleted (using ultracentrifugation as previously described [16]) FBS (Thermo Fisher Scientific, Waltham, MA, USA), 2 mM L-glutamine, and 1% penicillin/streptomycin (Solarbio, Beijing, P.R. China) at 5% CO_2_ and 37°C. The phenotype of BMSCs was determined using the antibodies CD34 fluorescein isothiocyanate (FITC) (555821, BD Biosciences, San Diego, CA, USA), CD45 FITC (555482, BD Biosciences), CD90 FITC (561969, BD Biosciences), and CD105 FITC (561443, BD Biosciences) via flow cytometry.

### 2.2. Cell Coculture

BMSCs and BEAS-2B cells were cocultured in Transwell permeable supports (Corning, Tewksbury, MA) using FBS-free DMEM. BEAS-2B cells were seeded into the lower chamber, whereas the BMSCs were seeded into the upper chamber. A 0.4 *μ*m porous membrane was used to separate these two cells. At 8 hr before coculture, the sEV inhibitor GW4869 (10 *μ*m, Sigma, CA, USA) dissolved in DMSO was added into the top chamber to reduce sEV release from BMSCs. Meanwhile, an miR-21-5p inhibitor and mimic were added into BMSCs in the upper chamber for 48 hr.

### 2.3. Enrichment and Characterization of Human BMSC-sEVs

The enrichment and characterization of human BMSC-sEVs were followed and performed as described in a previous publication [17]. Briefly, BMSCs were collected through centrifugation three times at 1,000, 2,000, and 10,000 g for 20 min each. The supernatants were then collected and condensed with a molecular weight cutoff of 100 kDa, layered over 30% sucrose/D2O (5 mL), subjected to ultracentrifugation at 100,000 *g* for 1 hr, and then diluted with PBS and condensed again. Thereafter, the purified sEVs were filtered through a 0.22-*μ*m pore filter (Millipore), after which transmission electron microscopy (TEM) (FEI Tecnai 12; Philips, Netherlands) was used to examine the morphology of BMSC-sEVs with 3% phosphotungstic acid solution staining. Furthermore, the enriched BMSC-sEVs were underwent nanoparticle tracking analysis (NTA) using a ZetaView machine with the software ZetaView 8.04.02 SP2. Meanwhile, two protein markers of sEVs, namely CD63 and CD81, were examined using western blotting, after which the enriched BMSC-sEVs were stored at −80°C for further analyses. The cells in the sEV treatment group were cultured with 200 *μ*g/mL of sEV protein in each well (1.5 mL media).

### 2.4. sEVs Uptake Assay

sEVs were stained with PKH67 (Sigma, USA) as described by Bang et al. [18]. After staining, the sEVs were diluted in PBS with 0.5 mL of Diluent C (Sigma, USA). The labeled sEVs were centrifuged at 100,000 × *g* for 1 hr and then collected and resuspended in PBS. For the sEV uptake assay, the labeled sEVs were added to the BEAS-2B cells. After 12 hr of coculture, BEAS-2B cells were stained using DAPI (Sigma, USA) and observed under a confocal microscope.

### 2.5. PCSK6 Expression Manipulation

Short interfering RNAs targeting PCSK6 (shPCSK61 : 5′- CCAGCAACAGGAAGUGAAATT-3′; shPCSK6-2, 5′-CCAACAGCAUCUACACCAUTT-3′; and shPCSK6-3 5′-GGAAGAUUACACAGCUCAATT-3′) and negative control short hairpin RNA (shRNA) (shNC, 5′-UUCUCCGAACGUGUCACGUTT-3′) were synthesized (Major Industrial Co., Ltd. Shanghai, China) and cloned into lentiviral plasmids (pLKO.1). For PCSK6 overexpression, we prepared a pLVX-puro lentiviral plasmid containing PCSK6 (NM_002570.5), cDNA (oePCSK6), and a mock plasmid as negative control (oeNC).

### 2.6. Cell Proliferation

Cell proliferation was assessed using the Cell Counting Kit-8 (CCK-8) assay (Signalway Antibody, Maryland, USA) after 0, 12, 24, and 48 hr of cell culture.

### 2.7. FITC-Dextran Permeability Assay and Transendothelial Electrical Resistance Measurement

Cells were supplemented with 1 mg/mL FITC-labeled dextran at 0 and 24 hr. After another 2 hr, the FITC signal intensity was measured using a microplate reader. The transendothelial electrical resistance (TEER) value was determined using a Millicell ERS-2 Voltohmmeter (Millipore, Bedford, MA).

### 2.8. Dual-Luciferase Reporter Gene Assay

Potential binding sites for miR-21-5p in the PCSK6 3′-untranslated region (UTR) were predicted using TargetScan and Starbase. According to the predictions, DNA fragments containing wild-type (WT) or MT sequences for miR-21-5p binding were synthesized and cloned into luciferase reporter vectors (pGL3-Basic). We introduced these constructs (namely, WT and Mut 3′-UTR), with an internal reporter plasmid and miR-21-5p mimic or inhibitor, into human BEAS-2B cells. Luciferase activities were measured 48 hr after transfection.

### 2.9. Real-Time Quantitative Polymerase Chain Reaction (RT-qPCR)

Total RNA was isolated from samples using TRIzol Reagent (Invitrogen, Waltham, MA, USA). The corresponding cDNAs were synthesized from the isolated RNAs using a kit (Thermo Fisher Scientific, Waltham, MA, USA). The conditions used for RT-qPCR were as follows: 95°C for 10 min, followed by 40 cycles of 95°C for 15 s, and 60°C for 45 s. The 2^−*ΔΔ*Ct^ method and normalization with GAPDH mRNA or U6 levels were used to calculate the relative expression. The primer sequences were as follows: miR-21-5p, F: 5′-GCGCGTAGCTTATCAGACTGA-3′, R: 5′-AGTGCAGGGTCCGAGGTATT-3′. PCSK6 F: 5′-TTCCTACGCCAGCTACGAC-3′, R: 5′-GCAACTTCTCCCGCACAAC-3′. GAPDH, F: 5′-AATCCCATCACCATCTTC-3′, R: 5′-AGGCTGTTGTC ATACTTC-3′. U6, F: 5′- AAAGCAAATCATCGGACGACC-3′, R: 5′- GTACAACACATTGTTTCCTCGGA-3′.

### 2.10. Protein Preparation and Quantification

The total protein of the samples was extracted using a protein extraction reagent (Thermo Fisher Scientific), and the protein concentration was initially measured using the bicinchoninic acid (BCA) method (Thermo Fisher Scientific). Briefly, the protein standard solution (in the BCA assay kit) was diluted using gradient dilution to calculate the standard curve, after which the protein sample (2 *μ*L) was added into the enzyme-linked immunosorbent assay plate with 18 *μ*L PBS and 160 *μ*L BCA working solution. After shaking the mixture, the sample was incubated for 30 min at 37°C. Thereafter, the sample was analyzed using the microplate reader (Thermo Fisher Scientific), and the absorbance of the solution at 562 nm was measured. The protein concentration was calculated according to the standard curve.

### 2.11. Western Blotting

Protein concentrations were initially measured using the BCA method (Thermo Fisher Scientific). Thereafter, total protein (25 *μ*g/well) was fractionated on 10% sodium dodecyl sulphate–polyacrylamide gel electrophoresis and transferred overnight to nitrocellulose membranes (Millipore, Billerica, MA, USA), which were then incubated sequentially with 5% nonfat dry milk at room temperature for 1 hr, primary antibodies at 4°C overnight, and then secondary antibody anti-mouse IgG (1 : 1,000; Beyotime, Shanghai, P.R. China) at 37°C for 1 hr. An enhanced chemiluminescence system (Tanon, Shanghai, P.R. China) was used to determine the protein levels. The primary antibodies used included the following: PCSK6 (Ab151562, Abcam, UK), CD63 (25682-1-AP, Proteintech, USA), CD81 (66866-1-Ig, Proteintech, USA), and GAPDH (60004-1-1 G, Proteintech, USA).

### 2.12. Statistical Analysis

Statistical analyses were conducted using GraphPad Prism 7.0 (La Jolla, CA, USA). The normality of the data has been assessed, and the data obeyed normal distribution characteristics. Data were presented as mean ± standard deviation (SD). From at least three samples or repeats. Comparisons between two groups were conducted using *t*-tests, whereas comparisons among multiple groups were conducted using a one-way analysis of variance with Tukey's post hoc test. A *p*-value of <0.05 indicated statistical significance. The normality of the data has been assessed, and the data obeyed normal distribution characteristic.

## 3. Results

### 3.1. Knockdown of PCSK6 Ameliorated LPS-Induced Inhibition of Proliferation and Increased Permeability in Human BEAS-2B Cells

**Figure 1:**
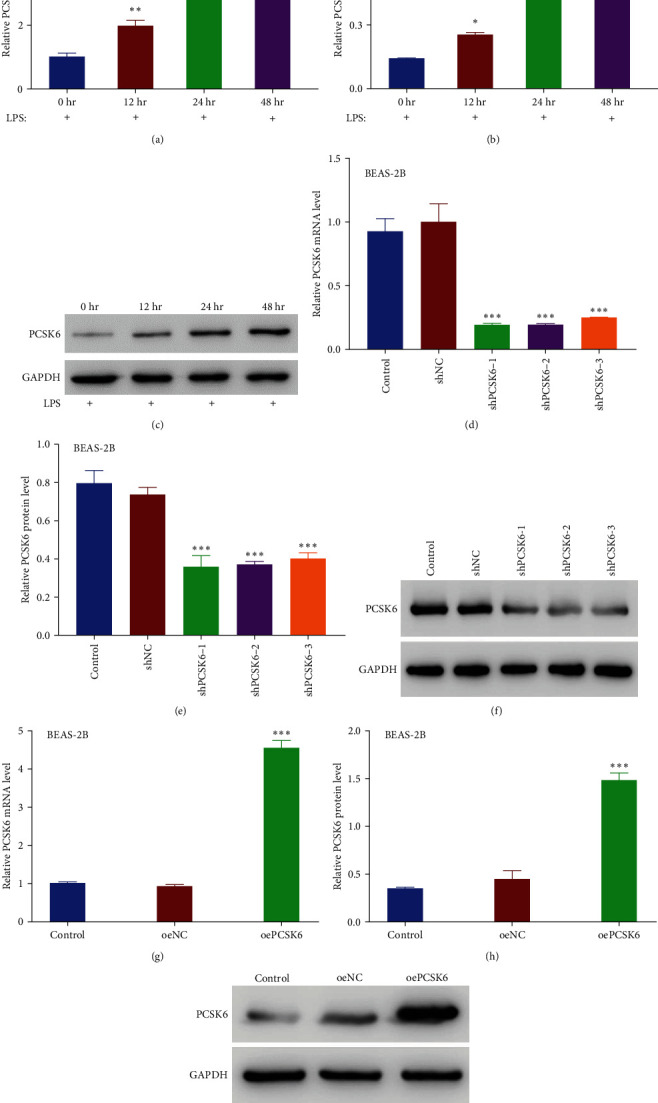
Effects of LPS stimulation on the mRNA and protein levels in human BEAS-2B cells, and the identification of PCSK6 shRNAs and overexpression vector. (a) RT-qPCR was used to examine the relative mRNA levels of PCSK6 at 0, 12, 24, and 48 hr in human BEAS-2B cells treated with LPS. (b, c) Western blotting was used to examine the protein levels of PCSK6 in human BEAS-2B cells treated with LPS at 0, 12, 24, and 48 hr. (d–f) PCSK6 shRNAs significantly suppressed the mRNA and protein levels of PCSK6 in human BEAS-2B cells. (g–i) Both mRNA and protein levels of PCSK6 were overexpressed in oePCSK6-transfected cells.  ^*∗*^*p* < 0.05,  ^*∗∗*^*p* < 0.01, and  ^*∗∗∗*^*p* < 0.001 vs. 0 hr/shNC/oeNC.

To establish a cell model of ALI, we used the BEAS-2B cell line, which originated from the human bronchial epithelium. BEAS-2B cells were cocultured with LPS, an important mediator of sepsis, which can induce ALI. Interestingly, LPS stimulation resulted in increased PCSK6 mRNA levels (Figure 1(a)), as well as elevated PCSK6 protein levels (Figures 1(b) and 1(c)) at 12 (196.07% and 179.06% of mRNA and protein levels at 0 hr, respectively), 24 (285.58% and 313.95% of mRNA and protein levels at 0 hr, respectively), and 48 hr (485.07% and 483.04% of mRNA and protein levels at 0 hr, respectively) after treatment, indicating upregulation of PCSK6 in the established ALI model. To understand the biological functions of PCSK6 in this context, shRNAs targeting human PCSK6 (shPCSK6-1, shPCSK6-2, and shPCSK6-3) and PCSK6 overexpression were generated and introduced into the cells. All three interfering RNAs greatly decreased the mRNA and protein levels of PCSK6, with the effects of shPCSK6-1 (19.86% and 48.34% of shNC mRNA and protein levels, respectively) and shPCSK6-2 (19.02% and 49.74% of shNC mRNA and protein levels, respectively) being slightly stronger than that of shPCSK6-3 (25.29% and 54.07% of shNC mRNA and protein levels, respectively) (Figure 1(d)–1(f)). Therefore, shPCSK6-1 and -2 were used for further experiments. Meanwhile, PCSK6 overexpression markedly increased mRNA and protein levels of PCSK6 (452.02% and 416.42% of oeNC mRNA and protein levels, respectively) (Figure 1(g)–1(i)).

**Figure 2:**
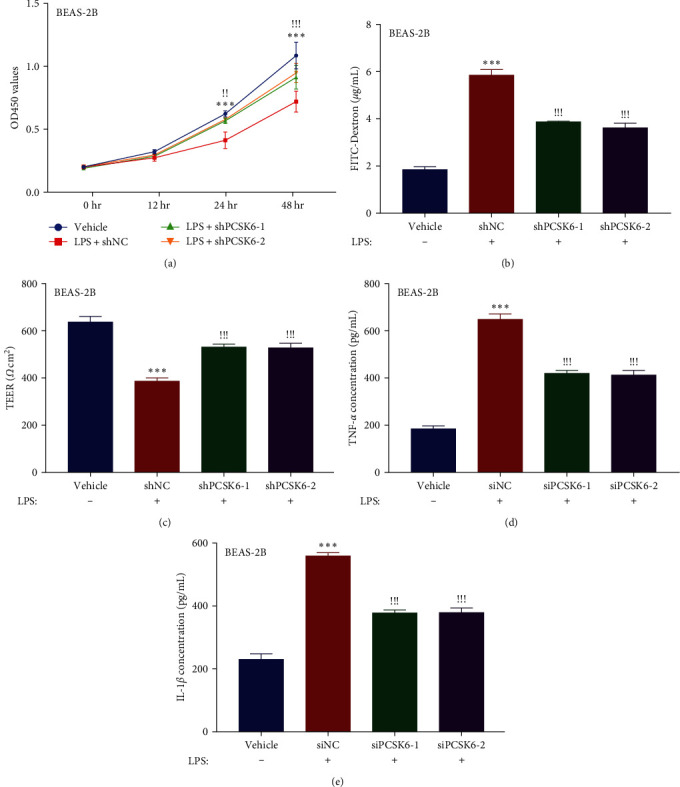
Knockdown of PCSK6 ameliorated LPS-induced inhibition of proliferation and increased permeability in human BEAS-2B cells. (a) shPCSK6-1 and shPCSK6-2 transfection significantly upregulated the proliferation of LPS-treated BEAS-2B cells. (b) shPCSK6-1 and shPCSK6-2 inhibited the extravasation of FITC-dextran from LPS-treated BEAS-2B cells. (c) Knockdown of PCSK6 increased the TEER value in LPS-treated BEAS-2B cells. (d, e) Knockdown of PCSK6 inhibited the secretion of TNF-*α* and IL-1*β* in LPS-treated BEAS-2B cells.  ^*∗*^ ^*∗*^ ^*∗*^*p* < 0.001 vs. vehicle. ^!!^*p* < 0.01, and ^!!!^*p* < 0.001 vs. shNC.

Consistent with previous literature [19], LPS stimulation significantly impeded the proliferation of BEAS-2B cells (inhibitory rate, 33.76% of vehicle, 48 hr after treatment). Conversely, silencing PCSK6 with either shPCSK6-1 (inhibitory rate, 15.95% of vehicle) or shPCSK6-2 (inhibitory rate, 12.73% of vehicle) effectively restored proliferative activity (Figure 2(a)). Moreover, LPS stimulation increased the permeability of BEAS-2B cells, as indicated by both TEER measurement (61.90% of vehicle, restored to 84.98% and 84.53%) and FITC-dextran permeability assay (318.74% of vehicle, restored to 209.25% and 186.84%), which were lowered by shPCSK6-1 and shPCSK6-2 (Figures 2(b) and 2(c)). Furthermore, PCSK6 silencing considerably reduced the secretion of two important pro-inflammatory cytokines, namely TNF-*α* (344.72% of vehicle, reduced to 231.23% and 218.76%) and IL-1*β* (228.78% of vehicle, reduced to 155.36% and 156.13%), which were upregulated by LPS (Figures 2(d) and 2(e)), suggesting the importance of PCSK6 in mediating LPS-induced inflammation.

### 3.2. PCSK6 Overexpression Disrupted the Therapeutic Effects of BMSC-sEVs in LPS-Treated BEAS-2B Cells

**Figure 3:**
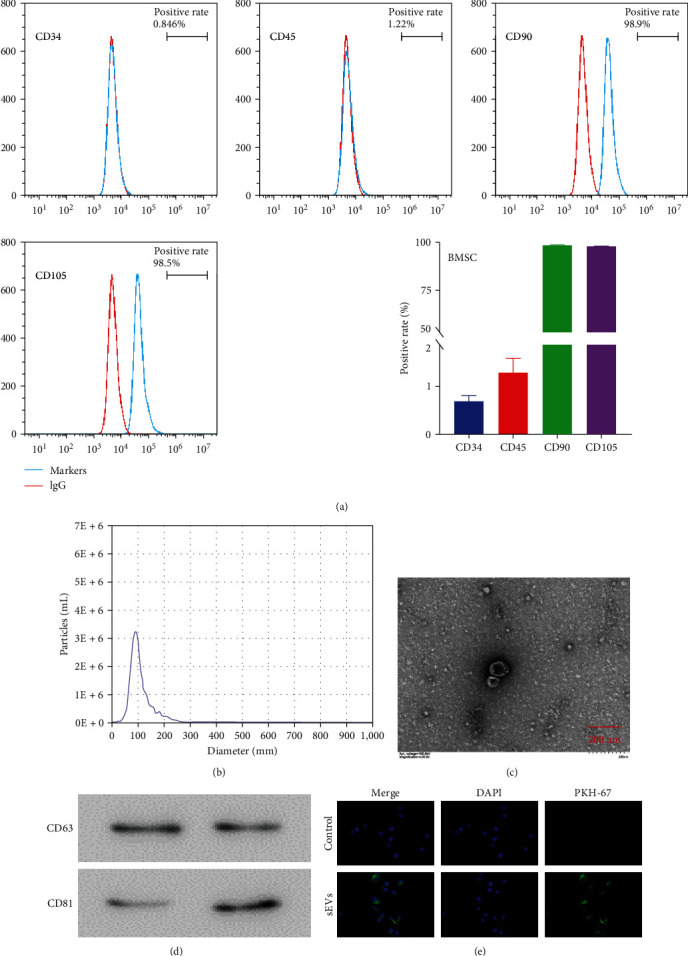
Identification of BMSC-sEV and sEV uptake in human BEAS-2B cells. (a) Identification of BMSCs using flow cytometry. The following antibodies were used to determine the BMSCs phenotype: CD34 FITC, CD45 FITC, CD90 FITC, and CD105 FITC. (b) Nanoparticle tracking analysis (NTA) results of enriched BMSC-sEVs. (c) TEM was used to examine the morphology of BMSC-sEVs. (d) Western blotting was performed to determine the protein contents of CD63 and CD81 in BMSC-sEVs. (e) The uptake of BMSC-sEVs labeled with PKH-67 by human BEAS-2B cells was determined using immunofluorescence.

Next, we studied the effects of BMSC-sEVs on LPS-treated BEAS-2B cells. To do this, we first examined the phenotype of BMSCs through flow cytometry using the antibodies CD34, CD45, CD90, and CD105. The positive rates were 0.796%, 1.215%, 98.75%, and 98.2%, respectively (Figure 3(a)). Thereafter, the sEVs were enriched from BMSCs and analyzed for their morphology, which was confirmed using NTA and TEM (Figures 3(b) and 3(c)). NTA results showed that the diameter of the main sEVs (96.1%) was 89.4 nm. In addition, two protein markers of sEVs, namely CD63 and CD81, were verified in multiple samples (two examples shown in Figure 3(d)), confirming the successful enrichment of BMSC-sEVs. By coculturing human BEAS-2B cells with fluorescently labeled BMSC-sEVs, we found that the BEAS-2B cells internalized the BMSC-sEVs (Figure 3(e)).

**Figure 4:**
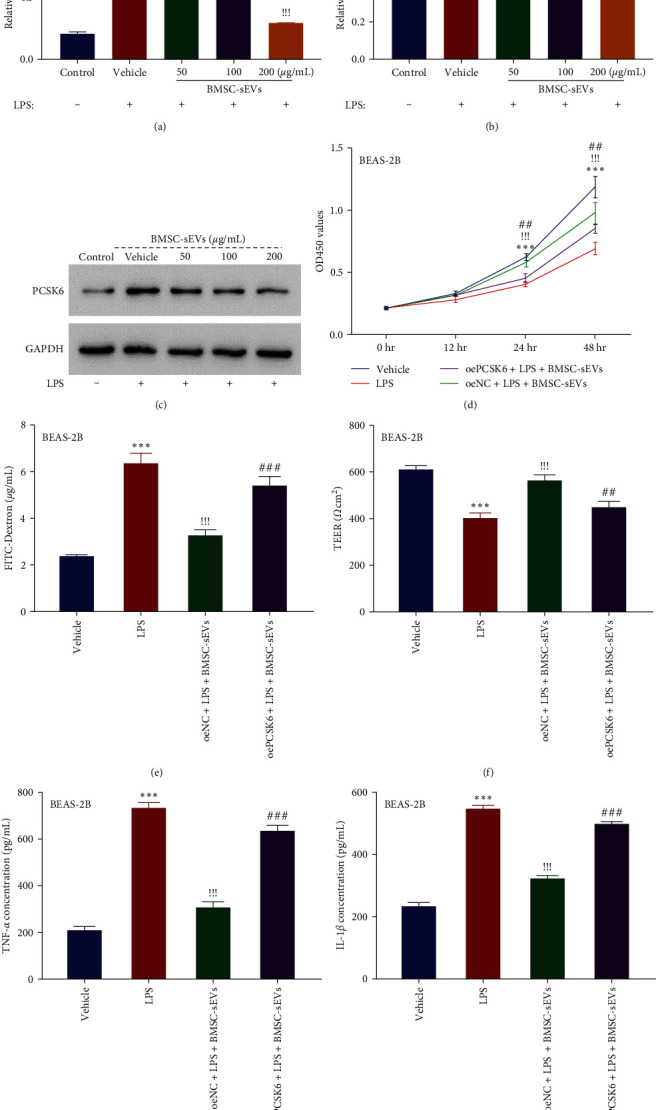
BMSC-sEVs ameliorated LPS-induced BEAS-2B cell injury by suppressing PCSK6 expression. (a–c) BMSC-sEVs inhibited the expression of PCSK6 in LPS-treated BEAS-2B in a dose-dependent manner.  ^*∗∗∗*^*p* < 0.001 vs. control ^!^*p* < 0.05, ^!!^*p* < 0.01, and ^!!!^*p* < 0.001 vs. LPS treatment group. (d) BMSC-derived sEVs ameliorated LPS-induced proliferation inhibition, but the effects were weakened by PCSK6 overexpression. (e) BMSC-derived sEVs reduced FITC-dextran of BEAS-2B cells exposed to LPS, but the effects were weakened by PCSK6 overexpression. (f) BMSC-derived sEVs enhanced the TEER value of BEAS-2B cells exposed to LPS, but the effects were weakened by PCSK6 overexpression. (g, h) BMSC-derived sEVs reduced TNF-*α* and IL-1*β* production in BEAS-2B cells exposed to LPS, but the effects were weakened by PCSK6 overexpression.  ^*∗∗∗*^*p* < 0.001 vs. vehicle. ^!!!^*p* < 0.001 vs. the LPS treatment group. ^##^*p* < 0.01, and ^###^*p* < 0.001 vs. oeNC + LPS + BMSC-sEVs.

Moreover, BEAS-2B cells treated with BMSC-sEVs at a dose of 50, 100, and 200 *μ*g/mL for 48 hr showed a decrease in the LPS-induced PCSK6 mRNA and protein upregulation (Figure 4(a)–4(c)) (482.16% and 244.57% of control mRNA and protein levels, respectively; mRNA levels restored to 390.88%, 284.73%, and 141.38%; protein levels restored to 204.94%, 176.61%, and 137.66%). Furthermore, the sEV dose of 200 *μ*g/mL was used in the subsequent experiment.

The following results showed that BEAS-2B cell proliferation decreased after LPS treatment for 48 hr (inhibitory rate, 41.54% of vehicle), and BMSC-sEVs treatment restored the cell viability (restored the inhibitory rate to 17.43%). However, the effects of BMSC-sEVs on cell proliferation were weakened by PCSK6 overexpression (Figure 4(d)) (increased the inhibitory rate to 28.11%). Furthermore, LPS stimulation increased the FITC-dextran (269.85% of vehicles) and decreased TEER (65.74% of vehicles) in BEAS-2B cells. We also found that BMSC-sEV treatment restored the alterations in permeability (restored FITC-dextran to 138.24% and TEER to 92.41%), and that PCSK6 overexpression abolished the effects of BMSC-sEVs (Figures 4(e) and 4(f)) (increased FITC-dextran to 229.14% and reduced TEER to 73.47%). Similarly, BMSC-sEVs treatment reduced the LPS-induced production of TNF-*α* (351.30% of vehicle, reduced to 147.64%) and IL-1*β* (236.46% of vehicle, reduced to 138.95%) (Figures 4(g) and 4(h)), whereas cotreatment with PCSK6 overexpression increased the TNF-*α* and IL-1*β* levels (Figures 4(g) and 4(h)).

### 3.3. BMSC-sEVs Inhibited PCSK6 Expression by Delivering miR-215p

**Figure 5:**
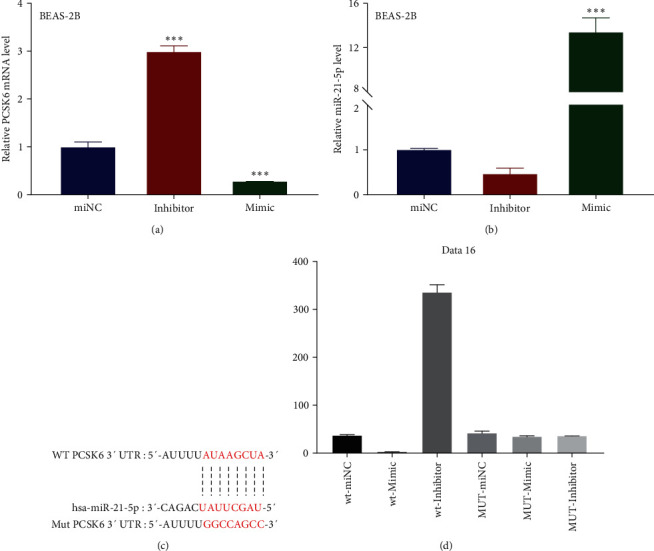
miR-21-5p regulated PCSK6 by binding to the 3′-UTR of PCSK6. (a) RT-qPCR was used to examine the expression of PCSK6 in BEAS-2B treated with miNC, an miR-21-5p inhibitor, and an miR-21-5p mimic for 48 hr. (b) RT-qPCR was used to examine the expression of miR-21-5p in BEAS-2B with the treatment of miNC, miR-21-5p inhibitor, and miR-21-5p mimic for 48 hr. (c) Illustration of the has-miR-21-5p-binding sequence in the 3′-UTR of WT PCSK6 and its change in the mutant PCSK6. (d) The luciferase activity in cells expressing WT and mutant PCSK6 was treated with miNC, an miR-21-5p inhibitor, and an miR-21-5p mimic.  ^*∗∗∗*^*p* < 0.001 vs. miNC.

The aforementioned results suggested that BMSC-sEVs might inhibit the effects of LPS by targeting PCSK6. Notably, miR-21-5p has been predicted to directly target PCSK6 mRNA [20]. To determine whether miR-21-5p is an important component in BMSC-sEVs responsible for PCSK6 inhibition, we initially transfected BEAS-2B cells with miR-21-5p mimic and inhibitor, after which the PCSK mRNA and miR-21-5p levels were determined using RT-qPCR at 48 hr after transfection. Our results indicated that the expression of PCSK mRNA was upregulated by the miR-21-5p inhibitor (298.50% of miNC) and decreased by the miR-21-5p mimic (Figure 5(a)) (29.11% of miNC). Meanwhile, the miR-21-5p mimic increased miR-21-5p levels (1336.57% of miNC). However, the miR-21-5p levels in the miR-21-5p inhibitor group showed no significant difference with that in the miNC (Figure 5(b)).

To confirm the regulation between miR-21-5p and PCSK6, we compared the sequence of has-miR-21-5p and the 3′-UTR of PCSK6 mRNA and predicted their potential binding sites using TargetScan [21] and Starbase. Thereafter, a mutation of the 3′-UTR of PCSK6 mRNA was designed to prevent miR-21-5p binding (Figure 5(c)). Luciferase reporter vectors containing sequences for the WT or mutant (MT) PCSK6 were then used to transfect BEAS-2B cells. After performing dual-luciferase reporter gene assay, we found that the luciferase activity in cells expressing WT PCSK6 was decreased by the miR-21-5p mimic (9.78% of miNC) and increased by miR-21-5p inhibitor (889.65% compared to miNC), whereas that in cells expressing MT PCSK6 was similar among all groups (Figure 5(d)), indicating that miR-215p directly binds to the 3′-UTR of PCSK6 mRNA to inhibit PCSK6 translation.

**Figure 6:**
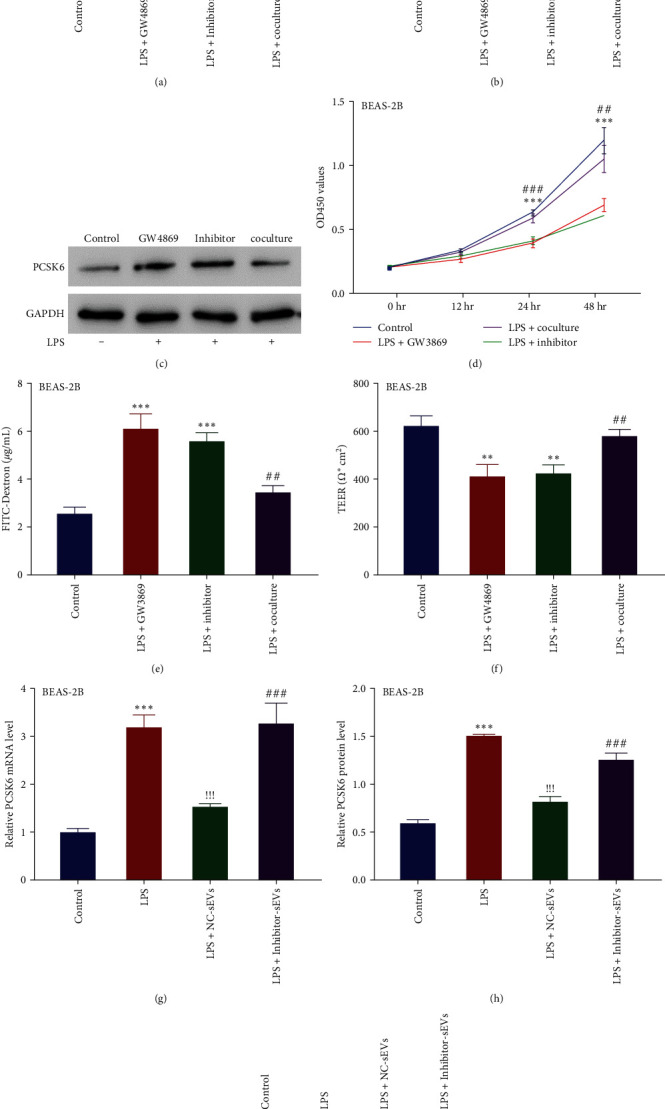
BMSC-sEVs ameliorated LPS-induced BEAS-2B cells injury by delivering miR-21-5p. (a–c) RT-qPCR and western blotting were used to examine the expression of PCSK6 in monocultured BEAS-2B cells (control), and BEAS-2B cells cocultured with GW4869- or miR-21-5p inhibitor-treated BMSCs. (d) The proliferation of monocultured BEAS-2B cells and BEAS-2B cells cocultured with GW4869- or miR-21-5p inhibitor treated-BMSCs. (e, f). The TEER value and FITC-dextran of monocultured BEAS-2B cells and BEAS-2B cells cocultured with GW4869- or miR-21-5p inhibitor treated-BMSCs.  ^*∗∗∗*^*p* < 0.001 vs. control. ^##^*p* < 0.001 vs. LPS + GW4869. (g–i) NC-sEVs decreased LPS-induced PCSK6 mRNA and protein expression, whereas the inhibitor-sEV promoted no significant change in the LPS treatment group.  ^*∗∗∗*^*p* < 0.001 vs. control. ^!!!^*p* < 0.001 vs. LPS. ^##^*p* < 0.01, and ^##^*p* < 0.001 vs. LPS + NC-sEVs.

Next, we determined the moderation effect of miR-21-5p on PCSK6 in an indirect cocultivation system. The exosome inhibitor GW4869 and an miR-21-5p inhibitor were added into the BMSCs in the upper chamber. Thereafter, LPS was added into the BEAS-2B cells in the lower chamber, after which cells were cultured for 48 hr. Our results indicated that PCSK6 mRNA and protein levels were increased in the GW4869 (304.26% and 364.44% of control mRNA and protein levels, respectively) and miR-21-5p inhibitor (351.28% and 387.02% of control mRNA and protein levels, respectively) treatment group. Meanwhile, the coculture group showed lower PCSK6 mRNA and protein levels (148.74% and 191.68% of control mRNA and protein levels, respectively) than that in the GW4869 and miR-21-5p inhibitor group (Figure 6(a)–6(c)). Furthermore, the proliferation of BEAS-2B cells was inhibited by GW4869 (inhibitory rate, 42.22% of control) and the miR-21-5p inhibitor (inhibitory rate, 48.92% of control), and coculture treatment restored cell viability (restored the inhibitory rate to 12.22%) (Figure 6(d)). Meanwhile, the cells in the GW4869 and miR-21-5p inhibitor groups showed increased FITC-dextran (239.70% and 218.46% of control) and decreased TEER (65.87% and 68.03% of control), and these parameters were restored in the coculture group (restored FITC-dextran to 135.63% and TEER to 93.02% of control) (Figures 6(e) and 6(f)).

Lastly, we determined the differences in the effects of BMSC-sEVs (NC-sEVs) and sEVs enriched from the miR-21-5p inhibitor (inhibitor-sEVs)-transfected BMSC cells on the mRNA and protein expression of PCSK6 in BEAS-2B cells. Our results showed that NC-sEVs decreased LPS-induced expression of PCSK6 mRNA (restored from 317.55% to 151.89% of control) and protein (restored from 254.93% to 137.26% of control), whereas the inhibitor-sEVs promoted no significant change in the LPS treatment group (Figures 6(g)–6(i)).

## 4. Discussion

In recent years, stem cell therapy has shown promising results in the treatment of lung injuries. In mouse disease models, MSC therapy successfully alleviated bleomycin-, hyperoxia-, ventilation- and sepsis-induced lung injuries [22]. In particular, MSC therapy inhibited bacterial growth, improved survival, and reduced organ dysfunction in models of sepsis-induced lung injury, demonstrating comprehensive therapeutic effects. Human clinical trials on the potential of stem cell therapy in treating ALI and acute respiratory distress syndrome have also started to emerge. Mechanistically, MSC therapy has been suggested to regulate lung injuries mainly through two pathways. The first pathway is the cell engraftment mechanism, in which MSCs can differentiate into healthy cells to replace the inflammatory and damaged somatic cells. The second pathway is the paracrine/endocrine mechanism, in which MSCs secrete soluble factors (e.g., anti-inflammatory cytokines) to the local microenvironment and regulate neighboring somatic cells. However, only recently have researchers realized that sEVs secreted by MSCs also contribute to ALI treatment via a paracrine/endocrine mechanism [23]. MSC-sEVs can release various proteins (e.g., Wnt4) and miRNAs (e.g., let-7b and miR-371b-5p), which target different signaling pathways to facilitate inflammation inhibition, fibrosis inhibition, and vascular reconstruction, among others [24]. Complementary to these discoveries, our study found that MSC-sEVs release another miRNA, namely miR-21-5p, to alleviate ALI via PCSK6 downregulation in bronchial epithelial cells. These results suggest the presence of a new mechanism underlying the therapeutic effects of MSC- sEVs on ALI, as well as establish a new signaling axis formed by miR-21-5p and PCSK6. Our work underscored the potential of using MSC-sEVs as a cell-free substitution for MSC transplantation in the treatment of ALI patients, which has the advantage of preventing transplant rejection.

PCSK6 is a proprotein convertase that cleaves protein precursors at the R-X-(K/R)-R motif to generate mature proteins, including ADAMTS-4 and -5, TGF-beta-related proteins, and von Willebrand factor. Studies have implicated PCSK6 in the modulation of cardiovascular function, tumor development, left-right patterning, etc. [25]. With regard to ALI, PCSK6 has been shown to facilitate carotid atherosclerosis [26] and inflammation in rheumatoid arthritis [27]. However, no previous study has investigated the relationship between PCSK6 and ALI. Here, we showed that LPS stimulation induced PCSK6, which promoted inflammation and apoptosis of bronchial epithelial cells and an increase in cell permeability. Silencing PCSK6 effectively inhibited the mentioned effects and restored the proliferative capability of the cells. These results suggest that PCSK6 is a major factor involved in ALI pathogenesis. At the molecular level, PCSK6 has been found to activate various pro-inflammatory signaling pathways in different cells, including the NF*κ*B, STAT3, and ERK1/2 pathways in synovial fibroblast-like cells [28] and the NLRP3 pathway in trophoblasts [29]. As such, investigating which signaling pathway(s) are responsible for the pro-inflammatory function of PCSK6 in bronchial epithelial cells in the context of ALI should provide deeper insights into ALI pathogenesis.

MiR-21-5p is an miRNA with versatile functions in vivo. It facilitates the progression of multiple types of cancers [30], stimulates extracellular matrix degradation and angiogenesis in temporomandibular joint osteoarthritis [31], and is involved in cardiovascular dysfunctions [32] and diabetes [33]. One study showed that miR-21-5p alleviated ALI in rats [15], the mechanism for which was found to be associated with alveolar type II epithelial cells. In addition, previous studies have shown that resveratrol could ameliorate pulmonary fibrosis by increasing miR-21-5p levels [34] and that miR-21-5p suppressed mitophagy to alleviate hyperoxia-induced ALI by directly targeting PGAM5 [35]. Another study demonstrated that AMSC-derived extracellular vesicles carrying miR-21-5p alleviated hyperoxia-induced lung injury [36]. These reports suggested that sEVs-miR-21-5p played a critical role in the repair process of lung injury. The current study has been the first to demonstrate the potential inhibitory effects of BMSC-derived sEVs-miR-215p in ALI in bronchial epithelial cells. Particularly, miR-21-5p facilitated cell growth and inhibited apoptosis, which is well aligned with previous characterizations of miR-21-5p functions [37]. However, unlike the molecular mechanisms found in previous works, like targeting KRIT1 or PI3K/AKT [37], the current study identified PCSK6 as a new target molecule. Identifying additional molecular targets of miR-21-5p involved in its mediation of ALI and their relevant signaling pathways will uncover more strategies for the design of next-generation precision medicine with higher specificity and less side effects. However, considering that some other miRNAs in the MSC-sEVs also provide protection against ALI [24], screening all miRNAs in MSC-sEVs to identify those that have inhibitory functions on ALI and consequently studying their respective mechanisms would be meaningful.

## 5. Conclusion

Our findings showed that PCSK6 played an important role in LPS-induced inflammation and apoptosis in bronchial epithelial cells, which is highly relevant to sepsis-induced ALI. BMSC-derived sEVs secreted miR-21-5p, which targeted PCSK6 to induce PCSK6 downregulation and subsequently alleviated inflammation and apoptosis in the cells. Our study underscores the potential of MSC-sEVs and miRNAs, particularly miR-21-5p, as promising strategies for treating ALI. However, although BMSC-derived sEVs-miR-21-5p play a critical role in the improvement of lung injury, more in-depth studies are still needed to determine the specific mechanisms underlying miR-21-5p and the downstream pathways of PCSK6.

## Data Availability

The datasets used and/or analyzed during the current study are available from the corresponding author upon reasonable request.
